# Bronchoalveolar Lavages Combined With Glucocorticoids in Management of Acute Exogenous Lipoid Pneumonia: 3 Case Reports

**DOI:** 10.1155/carm/4165969

**Published:** 2025-08-30

**Authors:** Xue Huang, Mingjun Wu, Qingliang Xue, Guowei Yu

**Affiliations:** ^1^Department of Respiratory and Critical Care Medicine, No. 940 Hospital of the of PLA Joint Logistics Support Force, Lanzhou, Gansu, China; ^2^Department of Medicine, Northwest Minzu University, Lanzhou, Gansu, China

**Keywords:** acute exogenous lipoid pneumonia, bronchoalveolar lavage, glucocorticoids, lipoid pneumonia

## Abstract

Exogenous lipoid pneumonia (ELP) is a rare disease with both acute and chronic forms. This paper primarily summarizes the diagnosis and treatment process of bronchoalveolar lavage combined with glucocorticoids treatment of acute ELP caused by aspiration of liquid hydrocarbons (e.g., kerosene and diesel fuel) at our hospital. Furthermore, the present study analyzes the advantages of bronchoalveolar lavage combined with glucocorticoids treatment of acute ELP.

## 1. Introduction

Exogenous lipoid pneumonia (ELP) is a rare disease caused by aspiration or inhalation of lipid-containing substances. Literature reported that the incidence is lower in adults (1.0%–2.5%) than in children (8.8%), probably because children are more prone to aspiration events than adults. ELP is usually categorized into acute and chronic. The most common causes of chronic ELP are the long-term use of oil-based laxatives and oily nasal drops and the long-term use of lubricants such as petroleum jelly during post-tracheotomy car [1–3]. In recent years, studies have suggested that the incidence of chronic ELP has increased among people who use “e-cigarettes.” The oil in e-cigarettes, when heated, produces aerosols containing a variety of chemicals, and prolonged exposure of the respiratory tract to these chemicals can lead to chronic ELP [4, 5]. The main cause of acute ELP is short-term inhalation of large quantities of oily substances (liquid hydrocarbons, e.g., kerosene and diesel fuel). It occurs most frequently in occupational exposures, such as actors in flamethrower performances, and dry-cleaners using hydrocarbon solvent aerosolizers [6], and the former has been referred to as “fire-eater's lung” [7]. In this paper, we report three cases of acute ELP admitted to our hospital and summarize the diagnosis and treatment processes in order to provide clinical reference for the early diagnosis and effective treatment of acute ELP.

## 2. Case 1

**Figure 1:**
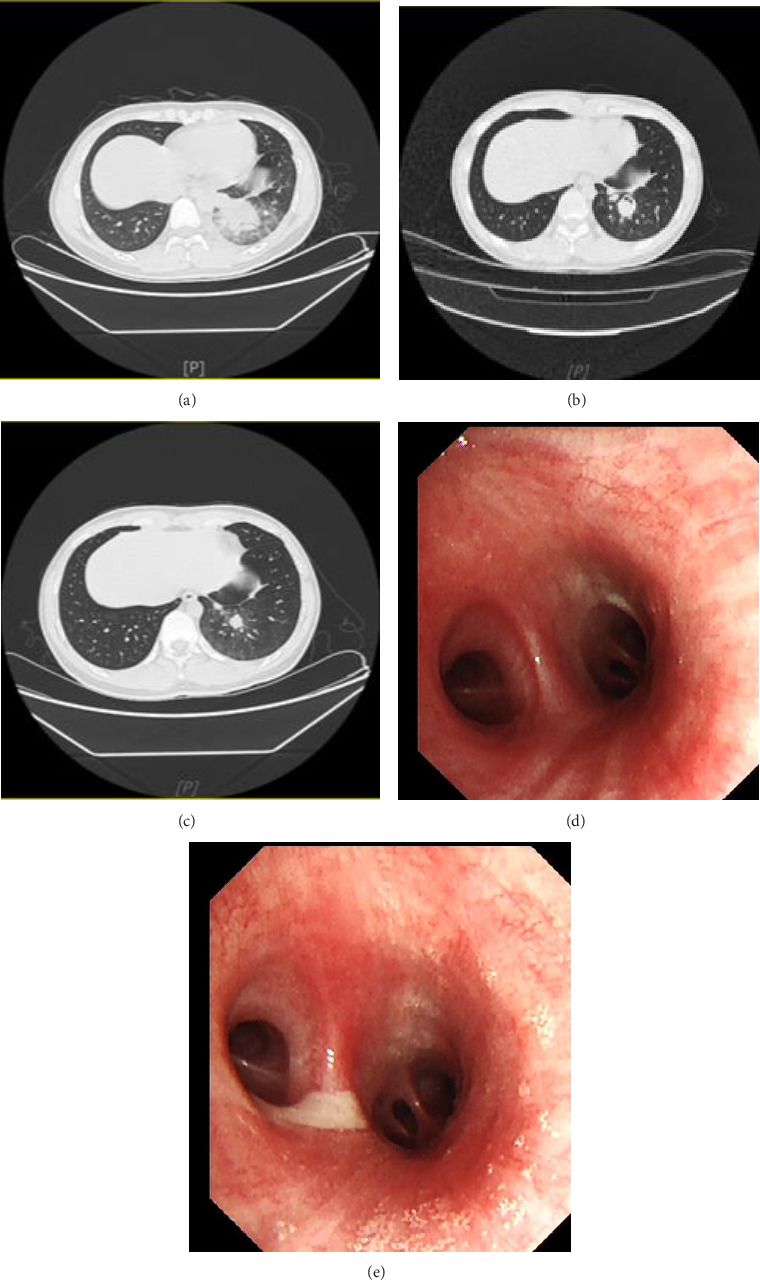
(a) July 17, 2024 chest CT showed massed solid and patchy shadows in the left lower lobe, limited thickened adhesions of the left pleura with pleural effusion. (b) July 23, 2024 chest CT showed multiple round-like hypodense shadows in the lower lobe of the left lung, and lung abscess formation was considered. The extent of the infection and the left pleural effusion were reduced compared to the previous CT. (c) July 29, 2024 chest CT showed significant resorption of a lesion in the lower lobe of the left lung compared to the previous CT. (d) July 22, 2024 bronchoscopy revealed reduced congestion and edema of the left lower lobe bronchial mucosa and disappearance of purulent secretions. (e) July 18, 2024 bronchoscopy revealed congestion and edema of the left lower lobe bronchial mucosa with purulent secretions.

The patient is a male fire-breathing performer, 21 years old, complaining of fever with cough and shortness of breath for 2 days. Last year, the patient accidently inhaled a large amount of fire-breathing oil (a type of kerosene) (about 15 mL) during a performance. Subsequently, he developed fever with cough, sputum, and shortness of breath and took “ibuprofen” orally to reduce fever, but the symptoms gradually aggravated. He was admitted to our hospital 5 days later. The chest CT showed that the left lower lobe was characterized by a mass of solid shadows and patchy shadows, with the left pleural effusion (Figure 1(a)). Blood gas analysis (FiO_2_ 21%): PO_2_ 70.06 mmHg, PCO_2_ 32.2 mmHg; blood routine: WBC 18.72 × 10^9^/L, N 89.1%; CRP 16.5 mg/dL; PCT 0.21 ng/mL; and IL-6 138.4 pg/mL. Combined with the above information, we considered the acute ELP and then administered “cefoperazone sulbactam 3 g ivgtt q12h” combined with “doxycycline 0.1 g po q12h” for antiinfection, and “prednisone 30 mg po qd” for anti-inflammation treatment. 1 day later, 4 days later, 7 days later, and 11 days after admission, the patient underwent bronchoalveolar lavage (BAL) of the basal segment of the left lower lobe. These all showed that the mucous membrane of the bronchial tubes was congested bilaterally, accompanied by secretion, which was obvious on the left side (Figures 1(d) and 1(e)). The lipid content of the BAL fluid was reduced step by step. After BAL, the patient's clinical symptoms gradually relieved. Repeating Chest CT (Figures 1(b) and 1(c)) showed that the left lower lobe lesion and left pleural effusion were gradually absorbed. He was discharged on oral prednisone.

## 3. Case 2

**Figure 2:**
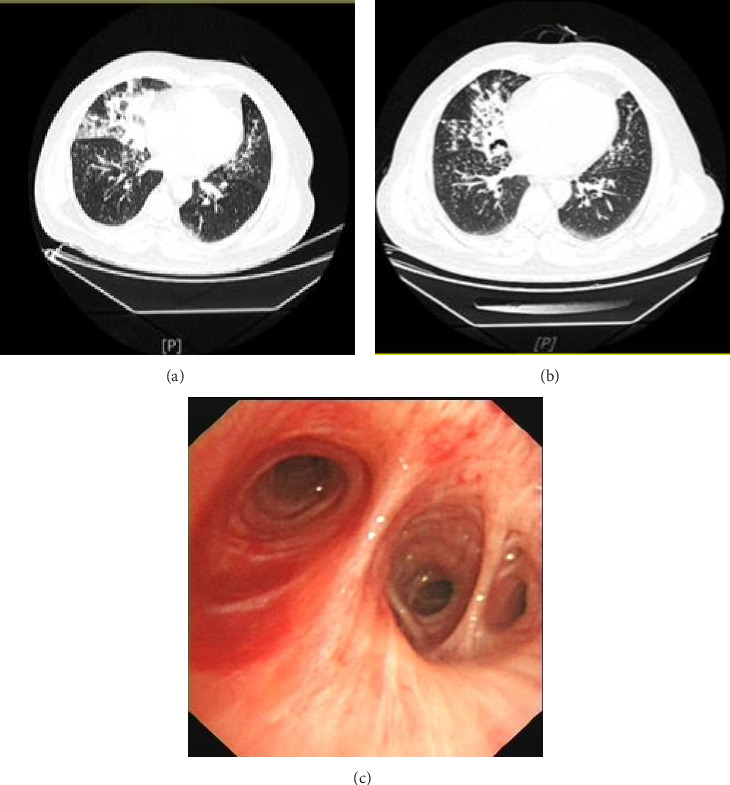
(a) August 18, 2022 chest CT showed multiple patchy solid shadows in both lungs. (b) August 26, 2022 chest CT showed a lesion in the middle lobe of the right lung that was significantly smaller than before. (c) August 18, 2022 bronchoscopy revealed bilateral bronchial mucosal congestion and edema, and a small amount of yellowish viscous secretion was visible in the lumen.

A 58-year-old male car mechanic presented with chest tightness, shortness of breath, cough, and sputum for 2 days. He accidently inhaled approximately 20 mL of diesel fuel during car repairs, followed by progressive respiratory symptoms. Initial chest CT at a local hospital revealed multiple lamellar shadows in both lungs, and anti-infective therapy was administered without improvement. The patient was transferred to our hospital 4 days after the inhalation incident. Repeat chest CT (Figure 2(a)) showed persistent lamellar solid shadows in both lungs, particularly in the right middle lobe and the lingual segment of the left upper lobe. Blood gas analysis (FiO_2_ 21%): PO_2_ 56.4 mmHg, PCO_2_ 34.7 mmHg; blood routine: WBC 10.25 × 10^9^/L, N 86.8%, CRP 30.5 mg/dL. Anti-inflammatory therapy with methylprednisolone (40 mg IV qd) was initiated on admission day. The next day, bronchoscopy revealed congested and edematous bilateral bronchial mucosa with yellow mucous secretions in the lumen (Figure 2(c)). BAL of the right middle lobe bronchus yielded fluid containing oil and grease, confirming the diagnosis of ELP. Under general anesthesia, BAL was performed on the right middle lobe and left upper lobe lingual bronchus, with dexamethasone (10 mg) infusion into each segment. The patient's chest tightness and dyspnea improved the day after the procedure. 8 days after admission, follow-up chest CT (Figure 2(b)) demonstrated significant resolution of the right middle lobe lesion. The patient was discharged with a tapering regimen of oral glucocorticoids (prednisone acetate 40 mg qd, decreasing by 5 mg weekly).

## 4. Case 3

**Figure 3:**
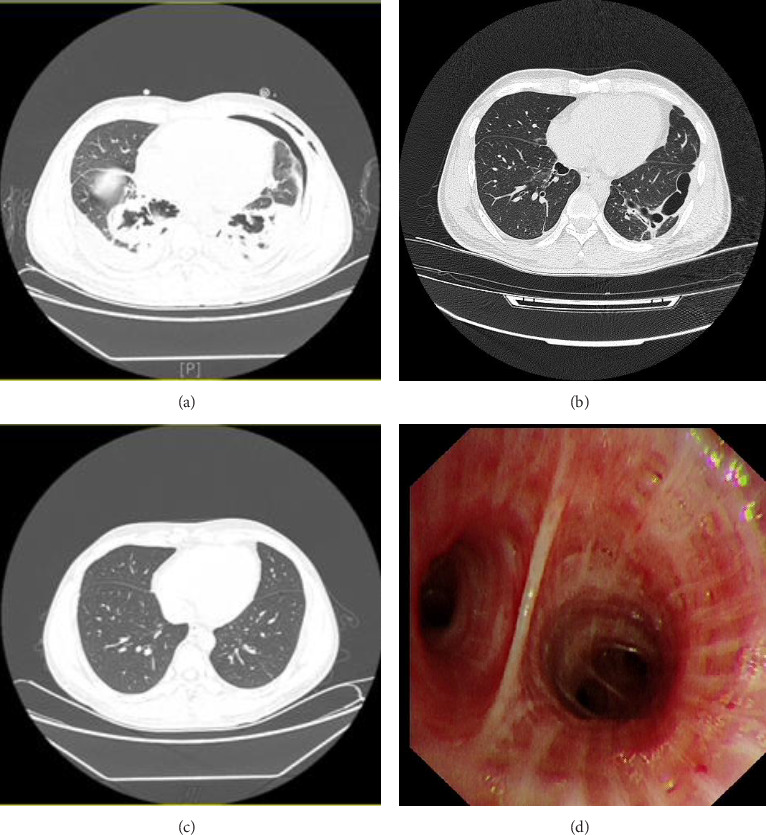
(a) July 14, 2022 chest CT showed a left-sided liquid pneumothorax, right-sided pleural effusion, bilateral lower lobe of lungs with exudation and cavity formation, bilateral pleural thickening and adhesions, and bilateral subcutaneous pneumatosis of the chest wall. (b) August 22, 2022 chest CT showed bilateral lower lobe exudates with cavity formation, left pneumothorax, bilateral pleural effusions, and subcutaneous pneumatosis of the left chest wall, which were less than before. (c) June 12, 2024 chest CT showed significantly better inflammatory lesions in the lower lobes of both lungs than before. (d) August 09, 2022 bronchoscopy revealed bilateral bronchial mucosal congestion and edema, predominantly in both lower lobes, and yellowish secretion was seen in the bronchus of the lower lobe of the left lung.

A patient accidently inhaled fire-breathing oil (≈20 mL) and was diagnosed with acute ELP 3 h later. Initial treatment with amoxicillin–clavulanate potassium (anti-infective) and methylprednisolone sodium succinate (anti-inflammatory) showed poor efficacy, leading to complications including lung abscess, pleural effusion, and pneumothorax. His condition deteriorated critically, prompting transfer to our hospital 11 days later. Chest CT revealed left-sided liquid pneumothorax, right-sided pleural effusion, bilateral lower lobe pulmonary exudation/cavity formation, bilateral pleural thickening/adhesions, and bilateral chest wall subcutaneous emphysema (Figure 3(a)). Blood gas analysis (FIO_2_ 40%) showed PO_2_ 88.2 mmHg and PCO_2_ 45 mmHg; laboratory findings included WBC 24.27 × 10^9^/L (neutrophil 87.8%), CRP > 100 mg/dL, PCT < 0.1 ng/mL, and IL-6 40.3 pg/mL. Diagnosis confirmed acute ELP with ARDS, left pneumothorax, and bilateral pleural effusion. Treatment included intravenous ceftriaxone (2 g daily), piperacillin–tazobactam (4.5 g q8h), vancomycin (1 g q12h), fluconazole (0.4 g daily), oral methylprednisolone (32 mg daily), and left chest tube drainage. On Day 24, CT indicated unresolved pneumothorax suggesting bronchopleural fistula, managed with continuous negative-pressure drainage (8 cmH_2_O). Poststabilization bronchoscopy revealed bilateral bronchial congestion/edema and yellowish secretions in the left lower lobe (Figure 3(d)). Subsequent CT (Figure 3(b)) showed resolved air leakage and clinical improvement, leading to discharge after 43 days. Final CT at 22 months postdischarge confirmed complete pulmonary resolution (Figure 3(c)).

## 5. Discussion

ELP is a type of aspiration pneumonia, and the common inhalants are liquid hydrocarbons, such as fire-breathing oil and jet fuel. These substances are characterized by high volatility, low surface tension, low viscosity, and insolubility in water. Moreover, they can diffuse rapidly in the airways, destroying the surfactant effectiveness and reducing lung compliance, and can also stimulate the inflammatory response of inflammatory cells in the alveoli, causing focal pneumonia and even the formation of granulomas and pulmonary fibrosis. Liquid hydrocarbons cannot be metabolized and mainly induce phagocytosis by macrophages and remain in macrophages for a long period of time, which lyses and then returns to the alveoli again [8], repeatedly causing tissue damage. The pathologic manifestation is the presence of a large number of lipid vacuoles and lipid-phagocytizing foam cells in the alveoli [9]. The clinical manifestations of acute ELP are often cough, sputum, fever, chest tightness, and shortness of breath with or without hemoptysis and malaise [10], which are not characteristic and do not require special treatment for these symptoms. Acute ELP can lead to severe hypoxemia and respiratory failure, and may progress to acute respiratory distress syndrome (ARDS) [11]. Therefore, early treatment can help to prevent the ARDS. Both patients in Case 1 and Case 2 of this article had cough, sputum, and shortness of breath as their main symptoms and their blood gas analysis showed hypoxia.

The diagnosis of ELP is challenging due to nonspecific clinical manifestations. The gold standard for the diagnosis of ELP is pathologic biopsy, but pathologic specimens are not readily available, and the final clinical diagnosis usually relies on a history of aspiration, chest CT, and histology of BAL fluid [12, 13]. The ELP localized mainly in the right middle lobe and both lower lobes, and the radiologic lesions may be displayed within a short period of time (< 24 h) after inhalants. Chest CT may show gross glassy clouding, pulmonary solid and paving stone signs, and irregular nodules [12, 14–16]. Without timely and effective treatment in the early stages, chest CT manifestations can further progress to more serious conditions such as lung abscess, pneumothorax, pleural effusion, cavitation, mediastinal emphysema, and bronchopleural fistula [18]. Because ELP is a rare disease with nonspecific clinical features, it is similar to the clinical manifestations of various infectious diseases and lung cancer and is easy to be misdiagnosed, especially when the CT shows a mass or nodule with lobular sign and burr sign, it is very easy to be misdiagnosed as lung cancer [10, 18]. Accurate diagnosis of ELP is very important to avoid unnecessary antibiotic treatment and invasive tests [19]. The patients of Cases 1 and 2 described in this article were finally diagnosed with acute ELP by taking a detailed history, clarifying the presence of a history of fire-breathing oil and diesel fuel aspiration, and combining with the chest CT manifestations, which provided a direction for early treatment.

There are no guidelines or expert consensus for the treatment of acute ELP, and some of the case reports have not been studied to define the optimal treatment program. Prevention of aspiration in high-risk groups is the key, and after the diagnosis is clear, segmental BAL combined with glucocorticoid therapy is a more advocated treatment option in clinical practice [3, 7, 8, 20]. Because liquid hydrocarbons cannot be metabolized and do not readily elicit a protective cough [21], they activate acute inflammatory responses of edema and interstitial fibrosis [22]. Timely removal of liquid hydrocarbons from the lungs by BAL and, at the same time, the application of glucocorticoids to limit or minimize the inflammatory response can reduce the damage to the alveolar and capillary endothelium caused by liquid hydrocarbons [23]. The damage caused by aspiration destroys the normal structure of the trachea, the defense function is impaired, and it is easy to secondary infection; therefore, infection should be treated with antibiotics as early as possible [24]. Most of the cases have been significantly improved by the above treatments [25, 26]. Cases 1 and 2 in this article both underwent segmental BAL with combined glucocorticoid treatment at our hospital in the early stage and achieved good therapeutic results. In severe acute ELP, surgical resection of severely involved and damaged lung lobes is feasible, especially when secondary to a large bacterial lung abscess, and surgical resection of the lesion may yield favorable results [27]. The literature suggests that with early active conservative treatment of acute ELP, the damaged lung tissue can recover well or even “restored to its original state” [28]. Case 3 of this article, although the segmental BAL, was not performed in the early stage of the first hospital, which resulted in serious complications. After being transferred to our hospital, continuous negative pressure chest drainage and segmental BAL, combined with oral glucocorticoids therapy, resulted in the disappearance of bilateral lung lesions and “restoration of the original state.” In summary, patients with acute ELP and early BAL combined with hormonal treatment can achieve better efficacy in the clinic, providing a direction for clinical diagnosis and treatment.

## Data Availability

The data supporting the findings of this study are publicly available.
